# Correlation between Sleep Disorders and Function in Children with Spastic Cerebral Palsy 

**Published:** 2019

**Authors:** Zahra GHORBANPOUR, Seyed Ali HOSSEINI, Nazila AKBARFAHIMI, Mehdi RAHGOZAR

**Affiliations:** 1 Occupational Therapy Department, University of Social Welfare and Rehabilitation Sciences, Tehran, Iran

**Keywords:** Sleep disorders, Function, Children, Cerebral palsy

## Abstract

**Objectives:**

The aim of the present study was to explain the correlation between sleep disorders and function in children with spastic cerebral palsy (4-12 year).

**Materials & Methods:**

This cross-sectional study was carried out on 62 children with spastic CP (8.98±1.46 yr) recruited from rehabilitation clinics of Tehran, Iran in 2017. The Activities Scale for Kids, The Sleep Disturbance Scale for Children and the cerebral palsy Quality of Life questionnaire for Children were utilized in this study. Data were analyzed using SPSS software.

**Results:**

Children with sleep disorder and arousal disorders had lower family health, lower quality of life and lower level of independence in their activities (*P*< 0.05).

**Conclusion:**

These results emphasize on the necessity of more attention about sleep disorders and family health problems in children with cerebral palsy.

## Introduction

Cerebral palsy (CP) is a group of non-progressive disorders in motor and posture occurring in developing fetal , that cause in activity and function limitation s (1) Children with CP have many problems in their life. The problems vary according to many reasons including the brain lesion area and CP type (2). These problems may impair the ability to perform daily life activities, as well as limiting social participation (3).

Considering to International Classification of Functioning, Disability & Health (ICF(, function refers to an total term that embraces both activities and participation (4). Function is often a significant drive of activities of daily living ability, quality of life development, engaging in personal care and social participation. Functional impairment can also cause cognitive, motor, communication and structure decline to accelerate (5). One of the problems that may affect the function of children with CP is sleep disorder.

There has been a focus in the literature on sleep disorders and writers stated that sufficient sleep has an important role in people’s health (6). Sleep plays vital role in brain function and systemic physiology of many body systems (6). This issue is more highlighted in children with CP as existing research on sleep disorders in children with CP reported that 23% of CP children have sleep disorders (with the mean age of 8 yr and 10 months), that is regarded as high when compared with 5% in the general population (7). This is probably due to several common factors in CP including muscle spasm, other types of musculoskeletal pain, and decreased ability to change body position during at nights which may all contribute to sleep difficulties and are also related to the primary motor impairment (8). Consequents of Sleep problems are: decrease motivation and concentration , cause in daytime sleepiness, mood disorders, memory deficit, and immunity decline (9) It may result not only in behavioral and neurological changes but also in families' psychical health (7). Sleep problems in children with motor disorders increase need for parental night-time attention. It is also one of the primary reasons for parent's stress who have children with chronic illnesses or are handicapped (10)

Despite the fact that function is an important issue in children with CP and that sleep disorders are probably an aggravating factor of function in children with CP, but the scientific evidence to support that is meager (11). 

Regardless the investigations about several problems in CP children separately on quality of life (QOL) or sleep problems or particularly motor dysfunction, (7, 11-17) there is not any registered study about sleep disorders and functional independence in CP children and more especially about the correlation between sleep disorders in spastic CP and functional independence in Iran. Therefore, the aim of the present study was to determine the correlation between sleep disorders and functional independence in children with spastic CP. By this, decreased sleep disorders lead to increased functional independence.

In the present investigation, according to ICF the functional independence was assessed based on having independence in daily life activities and quality of life.

## Materials & Methods

This descriptive study was conducted on 62 consecutive children with spastic CP (8.98±1.46 yr) recruited from Tehran rehabilitation clinics (Affiliated to the University of Social Welfare and Rehabilitation Science, Tehran, Iran) in 2017. 

The inclusion criteria were: age between 4-12 yr, child`s diagnosis of cerebral palsy by a neurologist, not using Anti-histamine, Melatonin, and Anti-seizure. The exclusion criterion was their unwillingness to participate in study. 

The Ethical Committee of the University of Social Welfare and Rehabilitation Science approved the study.

Subjects who participated in the study were explained about the study and the consent forms and related questionnaires for data collection were filled in a session lasted for one hour by the parents. For data collection, a demographic questionnaire was prepared for some basic information and the child’s level of cognition form was utilized, the form was a quick estimating form consisted of six questions about child's learning and playing ability compared to others. By evaluation of these questions, the researcher can classify child in three levels of <50, 50-70, 70< (18). Additionally, the Gross Motor Function Classification System (GMFCS E&R) (expanded and revised edition), was used to classify the children with CP according to their gross motor ability, limits, and need for assistive devices. This system contained five levels. Level I (the most independence) and level V (minimal independence) in motor function (19). Validity and reliability of the Persian version were approved (20). Manual Ability Classification System (MACS) was used to classify children with CP according to their fine motor ability. This system deals with how children with CP in 4-18 yr old use their hands for manipulating objects in activities of daily living (ADL). In this system, five levels have been defined. These levels are based on children’s need for getting help from others or adaptation (simplification) in performing manual tasks in ADLs. Level I indicate the best manual ability and level V indicates the fact that the child has no operative manual activity (21). validity and reliability of the Persian version were also approved (22).

**QOL Measurement**

QOL was assessed using the CP QOL-Child questionnaire. The CP QOL-Child is a condition-specific QOL questionnaire designed for children with CP to assess well-being rather than ill-being (23). It has two copies (self-report, caregiver report). The second copy was used (caregiver report for 4-12 yr old children) and it contained 66 items in 7 domains: social well-being and acceptance, functioning, participation, and physical health, emotional well-being, access to services, pain, and impact of disability and family health. Items response ranged from 1 (the most dissatisfied) to 9 (the most satisfied). The validity and reliability of this measure were obtained in Iran in a way that Cronbach's alpha ratio ranged from 0.61-0.87 and reliability ranged from 0.47-0.84 (24).

**Sleep Disorders** **Measurement**

Sleep disorders were assessed by The Sleep Disturbance Scale for Children (SDSC( questionnaire. The scale included 26 items on a Likert-type scale to assess 6 domains of sleep: disorder of initiating and maintaining sleep, sleep breathing disorder, disorder of arousal, sleep-wake transition disorder, excessive somnolence and sleep hyperhidrosis. The items response ranged from 1 (never) to 5 (always) and higher score indicates more acute sleep disturbance. The scale has been validated with youth populations aged 6-15 yr ( an internal consistency ranging from 0.71 to 0.79, test-retest reliability of 0.71 and a diagnostic accuracy of 0.91 (12, 25).

**Activities Scale for Kids (ASK) Measurement**

Finally, the Activities Scale for Kids (ASK) were used to assess activities independence in children with musculoskeletal disorders (aged 5-15 yr old). The scale included 30 items in 8 domains: personal care, dressing, transfer, locomotion, play, standing skill, stairs and other skills (26). The validity and reliability of this measure were obtained in Iran with Cronbach's alpha coefficient to be 0.997 (27).

**Statistical Analysis**

Data were analyzed using One-Sample Kolmogorov-Smirnov test for normality assessment, Spearman & Pearson for correlation analysis, One Way ANOVA, Man Whitney and Independent Sample *t*-test for average comparison and by SPSS software (Ver. 16, Chicago, IL, USA).

## Results

The study population consisted of 32 boys and 30 girls with the mean age of 8.98±1.46; range 6-12 yr old and 82.25% of them have sleep disorders. The demographic and clinical characteristics of study population are summarized in Table1.

**Table 1:** Demographic and clinical characteristics of study population

Variables		Number	Percent
Gender	Girl	30	48.4
	Boy	32	51.6
GMFCS^1^ levels	I.	14	22.6
	II.	18	29.0
	III	25	40.3
	IV.	5	8.1
Cognition level	<50	29	46.8
	50-70	25	40.3
	>70	8	12.9
Cp types	Hemiplegia	12	19.4
	Quadroplegia	33	53.2
	Diplegia	17	27.4
MACS^2^ levels	I.	7	11.3
	II.	21	33.9
	III	27	43.5
	IV.	7	11.3

1 .Gross Motor Function Classification System

2 .Manual Ability Classification System

To explain the relation between sleep disorders and gender, age, CP type, GMFCS levels, MACS levels and cognition levels, we compared the sleep disorders total score mean based on gender, age, CP type, GMFCS levels, MACS levels and cognition levels. According to the results sleep disorders total score was greater in girls compared to the boys and also sleep disorders total score was greater in level ΙΙ of MACS when compared to the other levels. Although sleep disorders total score was the greatest in level III in GMFCS, level III in cognition (>70) and hemiplegia in CP type there wasn’t any significant relation between sleep disorders total score with GMFCS levels, and cognition levels and CP type (Table 2).

**Table 2:** Sleep Disorders total score mean based on gender, age, CP type, GMFCS level and cognition level

Characteristics		Sleep disorders total score^4^	*P*-value
Gender^1^	Girl	53.7±14.06	0.032
	Boy	48.06±16.07	
GMFCS* levels^2^	I.	48.71±14.72	0.066
	II.	55.88±19.97	
	III	49.28±12.32	
	IV.	45.80±8.4	
MACS** levels^3^	I.	54.28±20.42	0.024
	II.	57.85±16.17	
	III	44.77±12.68	
	IV.	49.28±5.70	
Cognition level^2^	<50	50.41±11.58	0.077
	50-70	48.36±18.04	
	>70	59.75±16.36	
Cp types^3^	Hemiplegia	58.91±14.63	0.117
	Quadroplegia	48.45±15.13	
	Diplegia	49.58±14.89	

1 . Mann-Whitney u (comparison of sleep disorders total average)

2 . Kruskal-Wallis (comparison of sleep disorders total average)

3 . One way ANOVA (comparison of sleep disorders total average)

4 . Mean±SD

* Gross Motor Function Classification System

** Manual Ability Classification System

To explain the relationship between sleep disorders with activities independence, we analyzed the sleep disorders total score and activities total score using Spearman test. The relation was not significant (r=0.8 3). However, the comparison of the activities total score between children with sleep disorders and those without sleep disorders using independent *t*-test showed that activities independence was lower in children with sleep disorders than children without sleep disorders (Table 3).

**Table 3:** The correlation between sleep disorders and activities independence

		ASK total score^ 3^	*P*-value	R
Sleep disorder	Yes	60.6192±14.13663	0.24^1^	0.83^2^
	No	66.2682±15.84118		

1 . Independent samples test

2 . Pearson correlation

3 . Mean±SD

Finally, to explain the relationship between sleep disorders total score and QOL domains, we analyzed sleep disorders and QOL domains total score using Spearman test. There was not any significant relation but if we consider either sleep disorders domains one by one or QOL domains one by one, the family health can be significantly lower in children with higher disorder of arousal (Table 4). Moreover, the comparison of the QOL total score between children with sleep disorders and those without sleep disorders using independent *t*-test showed that the QOL total score mean was lower in children with sleep disorders (406.97±62.33) than children without sleep disorders (419.9±49016).

**Table 4:** The correlation between sleep disorders domains and sleep disorders total with quality of life domains

QOL domains*								
Sleep disorders total score^1^		SWB^1^	Function	Participation^1^	EWB1	ACC^1^	Pain	Family^1^
		0.129	0.467	0.872	0.256	0.256	0.893^1^	0.198
Sleep disorders domains**	DIMS	0.92	0.39	0.52	0.26	0.83	0.78^2^	0.31
	SBD	0.15	0.84	0.63	0.15	0.78	0.33^1^	0.86
	DA	0.74	0.79	0.36	0.74	0.29	0.99^1^	0.05
	SWT	0.21	0.14	0.16	0.44	0.63	0.55^2^	0.10
	DESS	0.58	0.95	0.43	0.19	0.96	0.94^2^	0.38
	SHY	0.54	0.63	0.37	0.54	0.24	0.61^1^	0.13

1 . Spearman's rho (Sig)

2 Pearson (Sig)

* Social well-being and acceptance (SWB), functioning (Function), Participation and physical health (Participation), Emotional well-being (EWB), Access to services (ACC), Pain and impact of disability (Pain) and Family health (Family).

** disorder of initiating and maintaining sleep (DIMS), sleep breathing disorder (SBD), disorder of arousal (DA), sleep-wake transition disorder (SWT), excessive somnolence (DESS) and sleep hyperhidrosis (SHY).

## Discussion

Sleep disruptions have substantial adverse short and long-term functional consequences (6). Our results showed that children with CP experience had significantly high frequency of sleep disorders compared with normal children. This difference is more likely due to cognitive, emotional and physical health problems in children with CP that can, in turn, increase the likelihood of poor sleep performance (28), and decrease the ability to change body position during the night which may all contribute to sleep disorders (8). 

One of the secondary aims of the present study was to compare the sleep disorders total score based on children’s demographic characteristics (gender, age, CP type, GMFCS levels, MACS levels and cognition levels). There was significant relation between age and sleep disorders, in agreement with some other studies (12, 29, 30). Sleep disorders in girls were higher than boys, in agreement with another study (12). The higher prevalence of depression and mood disruption in girls had effects on sleep quality and related to this result (31), disagreement with Cohen et al. study (32). Moreover, there was not a significant relation between sleep disorders total score and CP type, GMFCS levels and cognition levels. One probable explanation for the absence of significant relation is that all of our samples were spastic CP which have mostly the same problems. These results are in agreement with other studies that found no significant relation between sleep disorders and CP type (12), (29). Furthermore, there was not any significant relation between sleep disorders and GMFCS levels (7, 30).

The main purpose of the study was to compare the relation between sleep disorders and function, the quality of life and the activities (according to ICF), in children with CP. There was not any significant relation between them. However, sleep disorders total score was higher in children with low activities independence and low QOL, low function. Higher sleep quality leads to higher health condition and higher health condition causes better quality of life, better activities level and, therefore, better functional independence. More additionally, sleep disorders may result not only in behavioral and neurological changes (body structure and function) but also they change psychical health of families (environmental factor) that is probably an aggravating factor in terms of QOL of an individual with CP (environmental factor) (7, 8). According to ICF, all these personal and environmental factors can affect children’s health and functional condition (4). Moreover, if we consider sleep disorders one by one, or QOL one by one, we will found a significant relation between family health and disorder of arousal. Families' level of welfare and health certainly leads to the more deduction of child many problems. Disorder of arousal depends on many problems in daily life. When the child’s daily problems and mental disturbance increase because of families' health and welfare problems, he or she won't relax and consequently disorder of arousal will increase too. The results are in agreement with a study that found environmental factors and caregivers' behaviors to also contribute to children's sleep disorders reduction (15). 

One limitation to the present research was that we designed the study as a cross-sectional descriptive study, and the frequency of sleep disorders was very high and we did not test the two groups, those with sleep disorders and those without sleep disorders in equal members. However, this resolved by statistical analysis as possible as we could.

**In conclusion, **children with CP experienced high frequency of sleep disorders. Moreover, there was a relationship between sleep disorders and function in children with CP. The more sleep disorders score was high, the more function would be lower. Therefore, it emphasizes the necessity of more attention to sleep disorders in children with CP to improve treatment protocols about sleep disorders and subsequently function in children with CP.

It is recommended to design the study with cross-sectional case-control format about children with sleep disorders and those without sleep disorders with equal number of participants in each group. It is recommended to repeat the study in a large group of children with CP and its other types too.
